# Comparing perinatal outcomes in gestational and cystic‐fibrosis related diabetes

**DOI:** 10.1002/ijgo.70615

**Published:** 2025-11-28

**Authors:** Briana Clifton, Basra Osman, Katelyn M. Tessier, Kathleen S. Mahan, Amir Moheet, Sarah A. Wernimont

**Affiliations:** ^1^ Medical School University of Minnesota Minneapolis Minnesota USA; ^2^ Masonic Cancer Center, Biostatistics Core University of Minnesota Minneapolis Minnesota USA; ^3^ Division of Pulmonary Medicine, Department of Medicine University of Minnesota Minneapolis Minnesota USA; ^4^ Division of Endocrinology, Diabetes and Metabolism, Department of Medicine University of Minnesota Minneapolis Minnesota USA; ^5^ Division of Maternal‐Fetal Medicine, Department of Obstetrics, Gynecology and Women's Health University of Minnesota Minneapolis Minnesota USA

**Keywords:** cystic fibrosis, cystic fibrosis related diabetes, gestational diabetes, perinatal diabetes

Cystic fibrosis (CF) is caused by mutations in the cystic fibrosis transmembrane conductance regulator (CFTR) gene, and contributes to lifelong respiratory, gastrointestinal, reproductive and metabolic health concerns for affected individuals.^1^ As life expectancy continues to increase for those with CF, more individuals are choosing to experience pregnancy. Gestational diabetes (GDM) and pre‐existing cystic fibrosis‐related diabetes (CFRD) impact 18%–62% of pregnancies in individuals with CF.^1^ While GDM and CFRD can alter maternal metabolism and contribute to hyperglycemia and adverse perinatal outcomes,^2^, ^3^ it remains unclear if there are specific differences in clinical care needs and outcomes related to GDM compared to CFRD.

To compare outcomes in CFRD and GDM, we completed a retrospective cohort study from 2011 to 2023 of all singleton, non‐anomalous pregnancies affected by CF within a health system obstetric outcome database^4^ (this study was reviewed by the University of Minnesota IRB #STUDY00021243) Of note, only individuals that do not opt‐out of research are included in this IRB approved database (STUDY00012822, Wernimont PI). CFRD was diagnosed based on evidence of hyperglycemia prior to pregnancy, and GDM was diagnosed by two‐step OGTT during the second or third trimester in those without evidence of CFRD before pregnancy.^5^ Metabolic, pregnancy, and neonatal outcomes were compared between individuals with CFRD and GDM using Wilcoxon rank‐sum or Fisher exact tests as indicated.

A total of 31 individuals with CF were identified: 16 with CFRD, six with GDM, and nine without diabetes (Figure S1). Examining baseline characteristics, no differences in age, parity, or insurance status were detected between those with GDM and CFRD (Table 1). Additionally, no differences in baseline pulmonary function (by FEV_1_), CFTR modulator use, CF exacerbation hospitalization, pre‐pregnancy or third trimester weight were identified. Examining metabolic outcomes, individuals with CFRD had higher median hemoglobin A1c (HbA1c) compared to those with GDM during preconception (6.3 vs. 5.3, *P* = 0.005) and third trimester (6.0 vs. 5.1, *P* = 0.033) periods. While no one with GDM required insulin before or during pregnancy, those with CFRD used a median of 22.0 units daily prior to pregnancy, which decreased to 15 units in the third trimester, with a median increase of 0.5 units daily (Table 1, Figure S2). No differences in pregnancy‐induced hypertensive disorders or gestational age at birth were observed. Additionally, there were no significant differences in median birth weight or neonatal hypoglycemia.

**TABLE 1:** Baseline characteristics and outcomes.

	GDM (*N* = 6)	CFRD (*N* = 16)	*P* value^a^
Age, median (Q1, Q3)	32.8 (29.8, 37.2)	32.2 (28.4, 35.8)	0.384
Nulliparous, *n* (%)	4 (66.7%)	11 (68.8%)	>0.999
Public insurance, *n* (%)	0 (0.0%)	6 (37.5%)	0.113
Pre‐pregnancy FEV1 (%), median (Q1, Q3)	84.5 (72.8, 85.8)	72.0 (63.2, 88.8)	0.417
Hospitalizations during pregnancy, median (Q1, Q3)	1.0 (0.2, 1.0)	0.0 (0.0, 1.0)	0.394
Hospitalization events during pregnancy, *n* (%)			0.814
0	2 (33.3%)	9 (56.2%)	
1	3 (50.0%)	5 (31.2%)	
3	1 (16.7%)	2 (12.5%)	
CFTR modulators in pregnancy, *n* (%)	3 (50.0%)	8 (50.0%)	>0.999
Pre‐pregnancy weight (kg), median (Q1, Q3)	54.9 (54.3, 61.2)	57.4 (55.4, 64.4)	0.376
Third trimester weight (kg), median (Q1, Q3)	69.5 (161.1, 78.5)	71.2 (63.4, 76.0)	0.825
Pre‐pregnancy HbA1c (%), median (Q1, Q3)	5.3 (5.3, 5.5)	6.3 (5.7, 7.5)	0.005
Third trimester HbA1c^b^ (%), median (Q1, Q3)	5.1 (5.0, 5.2)	6.0 (5.5, 6.2)	0.033
Pre‐pregnancy insulin total daily dose^c^ (units), median (Q1, Q3)	0.0 (0.0, 0,0)	22.0 (11.5, 23.4)	0.005
Third trimester insulin total daily dose^d^ (units) median (Q1, Q3)	0.0 (0.0, 0.0)	15.0 (5.9, 31.9)	0.004
Pre to third trimester insulin total daily dose change^e^ (units), median (Q1, Q3)	0.0 (0.0, 0.0)	0.5 (−2.2, 8.7)	0.368
Pregnancy‐induced hypertension, *n* (%)	2 (33.3%)	4 (25.0%)	>0.999
Gestational age at birth, median (Q1, Q3)	37.6 (37.0, 38.8)	36.5 (35.4, 37.7)	0.076
Birth weight (g), median (Q1, Q3)	3215.0 (2919.8, 3460.0)	3189.4 (2601.1, 3434.0)	0.825
Neonatal hypoglycemia (<40 mg/dL), *n* (%)	4 (66.7%)	6 (37.5%)	0.348

Abbreviations: CFRD, cystic fibrosis‐related diabetes; CFTR, cystic fibrosis transmembrane conductance regulator; GDM, gestational diabetes; HbA1c, glycated hemoglobin.

^a^ *P* value is for Wilcoxon rank‐sum test for continuous variables, and Fisher exact test for categorical variables.

^b^ Third trimester HbA1c was missing for two individuals with GDM and two with CFRD.

^c^ Pre‐pregnancy insulin total daily dose was missing for three individuals with CFRD.

^d^ Third trimester insulin total daily dose was missing for two individuals with CFRD.

^e^ Change in total daily insulin dose was missing for four individuals with CFRD.

We found that individuals with CFRD had higher insulin requirements and HbA1c levels compared to those with GDM, although both groups had similar maternal and neonatal outcomes. While no individuals with GDM required insulin, the third‐trimester total daily insulin dose for individuals with CFRD increased from pre‐pregnancy levels, although it was less than might be expected based on weight‐based dosing in the general population.^6^ This suggests that CFRD is potentially metabolically distinct from other forms of perinatal diabetes. The strengths of this study include robust antenatal monitoring of pre‐pregnancy glycemia in a dedicated center and similar baseline characteristics among those with GDM and CFRD. Limitations include overall small numbers of patients and its single‐system design, including its dedicated CF care team, which may not be representative of care models for all individuals. However, this study demonstrates similar outcomes between individuals with GDM and CFRD and demonstrates lower‐than‐expected insulin needs during pregnancy in both groups.

## AUTHOR CONTRIBUTIONS

B.C. helped design the study, collected and analyzed the data, and reviewed the manuscript. B.O. collected and analyzed the data and reviewed the manuscript. K.M.T. completed the statistical analyses and reviewed the manuscript. K.S.M. and A.M. analyzed the data and reviewed the manuscript. S.A.W. designed the study, analyzed the data and wrote the manuscript.

## CONFLICT OF INTEREST STATEMENT

There are no competing conflicts of interest.

## Supporting information


**Figure S1.** Flow chart of individuals identified with cystic fibrosis and diabetes (CFRD and GDM).
**Figure S2.** Graph of individual total daily insulin dose prior to pregnancy and in the third trimester.

## Data Availability

Data available on request from the authors.
