# Supplementary figures and images for: Comparing perinatal outcomes in gestational and cystic‐fibrosis related diabetes
Source: Int J Gynaecol Obstet. 2025 Nov 28;173(2):1139–41. doi: 10.1002/ijgo.70615 (PMC13094672; doi:10.1002/ijgo.70615)

**Supplemental Figure 1:**


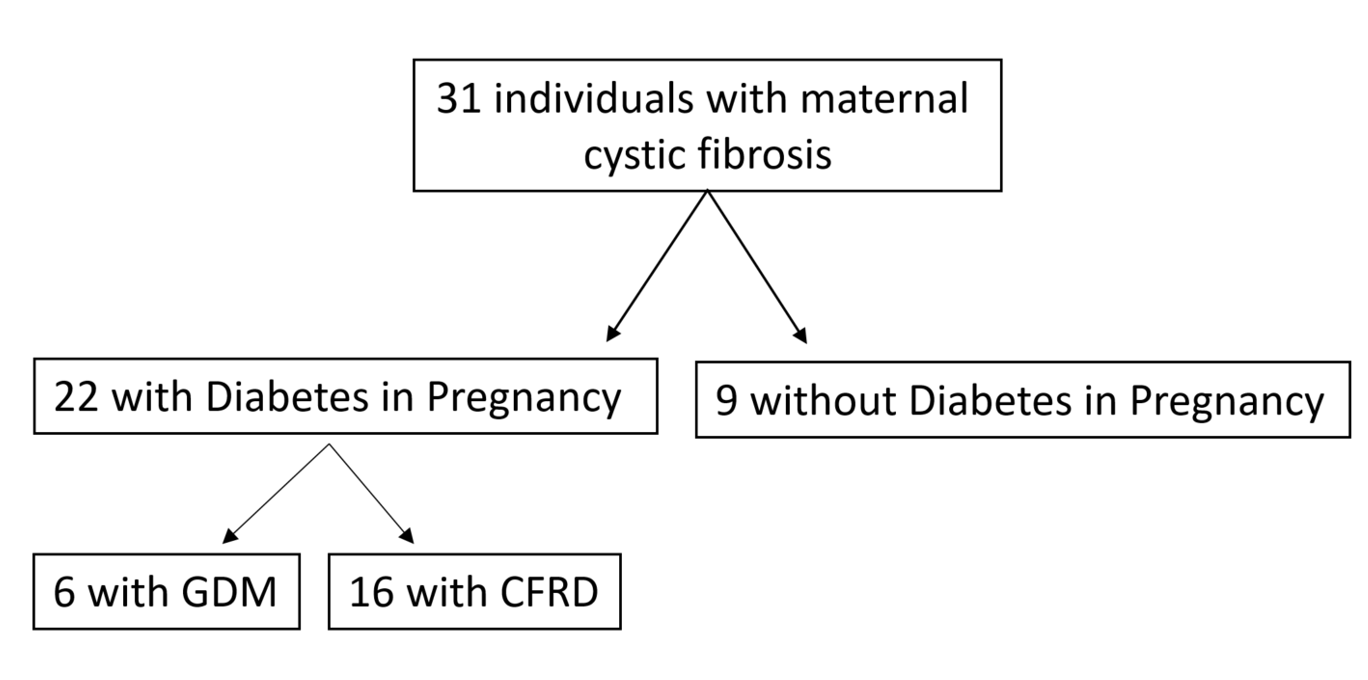


**Supplemental Figure 2:**


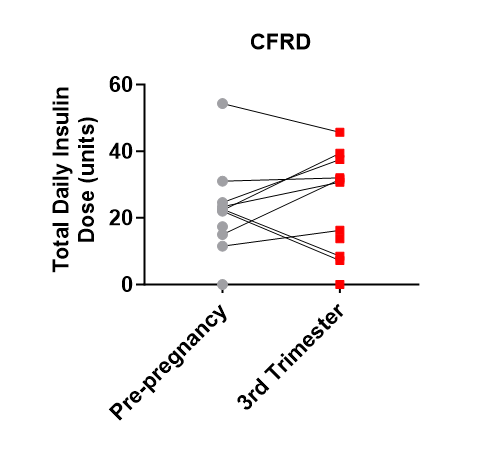

Supplement: Supplementary file 1 — Figure S1. Flow chart of individuals identified with cystic fibrosis and diabetes (CFRD and GDM). Figure S2. Graph of individual total daily insulin dose prior to pregnancy and in the third trimester. [file IJGO-173-1139-s001.docx]
